# Cetylpyridinium Chloride (CPC) Exhibits Potent, Rapid Activity Against Influenza Viruses *in vitro* and *in vivo*

**DOI:** 10.20411/pai.v2i2.200

**Published:** 2017-06-26

**Authors:** Daniel L. Popkin, Sarah Zilka, Matthew Dimaano, Hisashi Fujioka, Cristina Rackley, Robert Salata, Alexis Griffith, Pranab K. Mukherjee, Mahmoud A. Ghannoum, Frank Esper

**Affiliations:** 1 Department of Dermatology, University Hospitals Cleveland Medical Center and Case Western Reserve University, Cleveland, Ohio; 2 Center for Medical Mycology, Department of Dermatology, University Hospitals Cleveland Medical Center and Case Western Reserve University, Cleveland, Ohio; 3 Department of Medicine, The University of Chicago, Chicago, Illinois; 4 Electron Microscopy Core Facility, Case Western Reserve University School of Medicine; 5 Hathaway Brown Science Research and Engineering Program, Cleveland, Ohio; 6 Division of Infectious Diseases and HIV Medicine, University Hospitals Cleveland Medical Center and Case Western Reserve University, Cleveland, Ohio; 7 Division of Pediatric Infectious Diseases, University Hospitals Cleveland Medical Center and Case Western Reserve University, Cleveland, Ohio

**Keywords:** Cetylpyridinium Chloride, CPC, Influenza, Respiratory tract illness, respiratory virus, quaternary ammonium compound

## Abstract

**Background:**

There is a continued need for strategies to prevent influenza. While cetylpyridinium chloride (CPC), a broad-spectrum antimicrobial agent, has an extensive antimicrobial spectrum, its ability to affect respiratory viruses has not been studied in detail.

**Objectives:**

Here, we evaluate the ability of CPC to disrupt influenza viruses *in vitro* and *in vivo*.

**Methods:**

The virucidal activity of CPC was evaluated against susceptible and oseltamivir- resistant strains of influenza viruses. The effective virucidal concentration (EC) of CPC was determined using a hemagglutination assay and tissue culture infective dose assay. The effect of CPC on viral envelope morphology and ultrastructure was evaluated using transmission electron microscopy (TEM). The ability of influenza virus to develop resistance was evaluated after multiple passaging in sub-inhibitory concentrations of CPC. Finally, the efficacy of CPC in formulation to prevent and treat influenza infection was evaluated using the PR8 murine influenza model.

**Results:**

The virucidal effect of CPC occurred within 10 minutes, with mean EC_50_ and EC_2log_ ranging between 5 to 20 μg/mL, for most strains of influenza tested regardless of type and resistance to oseltamivir. Examinations using TEM showed that CPC disrupted the integrity of the viral envelope and its morphology. Influenza viruses demonstrated no resistance to CPC despite prolonged exposure. Treated mice exhibited significantly increased survival and maintained body weight compared to untreated mice.

**Conclusions:**

The antimicrobial agent CPC possesses virucidal activity against susceptible and resistant strains of influenza virus by targeting and disrupting the viral envelope. Substantial virucidal activity is seen even at very low concentrations of CPC without development of resistance. Moreover, CPC in formulation reduces influenza-associated mortality and morbidity *in vivo*.

## INTRODUCTION

Influenza is responsible for substantial morbidity and mortality worldwide [1–5]. Globally, influenza is estimated to adversely affect up to 5% to 10% of adults and 20% to 30% of children each year [6]. Annual influenza epidemics in the United States result in nearly 600,000 life-years lost, 3.1 million days of hospitalization, and 31.4 million outpatient visits [7]; in addition, the total economic burden of influenza exceeds $80 billion. Current prevention strategies for influenza are dependent on the use of anti-influenza medications and vaccines.

Neuraminidase inhibitors (NAIs, eg oseltamivir) are approved in the United States to prevent and treat influenza [8, 9]. However, the use of antiviral medications for pre-exposure prophylaxis has a very limited role and is generally not recommended for the majority of the population [10]. Also, NAIs confer only modest decreases in symptom duration for individuals presenting with uncomplicated illness [11–13], and this treatment suffers from the selection of resistant strains, adverse effects, and high cost [14–16]. While the most effective way to prevent influenza disease and its severe outcomes is by vaccination, current coverage estimates are well below the *Healthy People 2020* goal of 70% [17, 18]. Additionally, vaccine/strain mismatch can result in low vaccine efficacy [19–22]. Vaccines may also be contraindicated, unavailable, less effective in immuno-compromised individuals, and suffer from perceived risks leading to “vaccine hesitancy” [23, 24]. Therefore, the development of effective novel strategies to prevent and treat influenza disease is a significant unmet need.

Cetylpyridinium chloride (CPC) has been used for decades against a variety of pathogens [25–28], and it disrupts the microbial lipid bilayer through physicochemical interactions, a mechanism that is unlikely to be affected by mutations in addition to being pathogen independent [29]. For this reason, CPC and other quaternary ammonium compounds are commonly employed in the prevention of bacterial and fungal infections within healthcare settings. Yet there is little evidence demonstrating their effectiveness against respiratory viruses. Here, we evaluated CPC efficacy against the prototypical respiratory influenza virus demonstrating: (1) direct virucidal activity against influenza, (2) rapid activity following exposure, (3) viral ultrastructure disruption, (4) absence of influenza resistance following prolonged exposure, and (5) prevention and treatment of influenza infection in a murine model.

## MATERIALS AND METHODS

**Viral Strains:** Influenza strains were obtained from national influenza repositories: influenza A/Victoria/3/1975 (H3N2), A/Virginia/1/2009 (H1N1), B/Lee/40, oseltamivir-resistant A/ California/08/2009 (H1N1)pdm09 and B/Memphis/20/1996 (corresponding ATCC numbers: VR-822, VR-1736, VR-1535, IRR# FR-202, IRR# FR-486), and a clinical strain of influenza A (H1N1pdm09) virus which was propagated from a patient sample hereafter referred to as ‘isolate 40'. Both oseltamivir-resistant strains contain the NA H275Y substitution. Influenza A virus (strain A/Puerto Rico/8/1934 H1N1) was obtained from ATCC and propagated in chicken eggs.

**Cytotoxic Concentration (CC_50_) of CPC:** Madin Darby canine kidney cells (MDCK, ATCC number CCL-34) were cultured at 37°C and in 5% CO_2_ to 90% confluence in DMEM supplemented with penicillin/streptomycin, L-glutamine, and 10% fetal calf serum (Gibco, N.Y., USA). Increasing concentrations of CPC (10μg/mL to 250μg/ml, Sigma-Aldrich, St. Louis, MO) diluted in phosphate buffered saline (PBS) were added to media and incubated at room temperature for 10 minutes. Following exposure, cells were washed 3 times in DMEM to remove residual CPC. Complete media was then reapplied and cells were returned to 37°C, 5% CO_2_. Viability was quantitated by neutral red uptake at 48 hours as previously described [30]. Toxicity data were used to determine the 50% cytotoxic concentration (CC_50,_ Prism; v6.0 software).

**Influenza Cell Culture:** Growth of the influenza virus was performed as previously described [31]. Briefly, MDCK were grown to 90% confluence followed by inoculation with influenza virus at a multiplicity of infection (MOI) of 0.1 for 1 hour. The inoculum was then removed and 500 μL of optiMEM (Sigma-Aldrich, St Louis, MO)] was applied. Infected cells were grown at 32°C for 72 hours at 5% CO_2_. Tissue Culture Infective Dose 50% (TCID_50_) analysis was performed as detailed elsewhere [32]. The TCID_50_ was defined as the amount of virus required to produce a cytopathic effect in 50% of culture wells. The cytopathic effect was defined as focal rounding, degenerative changes, and detachment of cell monolayers.

**Hemagglutination Assay:** Hemagglutination assays were performed as described previously [33]. Briefly, 25 μL of viral sample was added to 25 μL of CPC diluted in PBS and subsequently incubated at room temperature for 5 minutes. Serial 2-fold dilutions were mixed with 50 μL of 0.5% suspension of chicken RBCs (Lampire Biological Laboratories, Pipersville PA) and incubated at room temperature for 30 minutes. Data were then presented as the percentage reduction in HA titer. The RBCs were exposed to CPC only to ensure CPC did not have a direct lysing effect on RBCs. Hemagglutination titers were determined as an end point dilution where no pellet was observed. The concentration of chicken RBCs was standardized using a hemocytometer.

**Transmission electron microscopy (TEM)**: Influenza virus (B/Lee/40) was chosen as a prototypical influenza virus and was treated with 50 μg/mL CPC for 5 minutes. A CPC concentration of 50 μg/mL was employed to ensure adequate visualization of CPC activity on TEM analysis while still remaining below CC_50_ toxicity levels. Fifty microliters of sample were placed on glass slides and formvar/carbon -coated TEM nickel grids (400 mesh) were placed over the samples face down for 1 minute. Influenza virus treated with PBS only was used as a sham control. Next, the grid was removed, blotted with filter paper and exposed to 2.0% uranyl acetate solution for an additional 1 minute. Excess uranyl acetate was removed, grids were air-dried, and examined under an FEI Tecnai Spirit (T12) electron microscope (TEI, Hillsboro, Oregon). Images were captured using a Gatan US4000 4kx4k CCD camera. The reviewer of TEM analysis was masked to treatment vs control slides.

**Influenza Nucleoprotein (NP) ELISA:** Samples were adsorbed overnight at 4°C in Costar 96-well plates. Wells were washed with PBS and 0.1% Tween 20-PBS, followed by blocking with BSA (1 g BSA in 50 ml PBS). Next, wells were exposed to mouse anti-influenza B NP antibody (diluted 1:1000, ABCAM) for 2 hours followed by secondary horseradish peroxidase-conjugated goat anti-mouse antibody (diluted 1:10,000, ABCAM) for 2 hours and finally tetramethylbenzi-dine (TMB) ELISA substrate (ABCAM) for 15 minutes; all incubations were performed at 37°**C**. Washes were performed between each incubation and reactions were terminated with ELISA stopping solution. Absorbance at 450 nm was quantified with a Biorad microplate reader. OD values were normalized with unexposed controls.

**Influenza challenge and evaluation of CPC in mice:** Wildtype mice (C57/BL6, Jackson Labs, Bar Harbor, ME) were infected intranasally (LD_50_, 8.0 x 10^3^ pfu/mouse) with the mouse-adapted influenza strain A/Puerto Rico/8/1934 H1N1 (PR8, ATCC). Viral stocks were propagated in embryonated chicken eggs and titered by hemagglutination assay as described above. Viral stocks were then titered *in vivo* to determine the LD_50_. The clinical formulation of CPC (ARMS-1 formulated with glycerine and xanthan gum for human use [34]) was given orally. To determine the prophylactic efficacy of ARMS-I, mice were treated with ARMS-1 (5 μL, applied orally) 15 minutes before the challenge and then twice a day for 5 consecutive days. Results were compared with oseltamivir phosphate (1 mg in PBS, applied IP) twice a day for 5 consecutive days or phosphate buffered saline (PBS) alone. Animals were observed daily for general appearance and body weight (calculated as percentage of initial weight on day 0). For assessment of therapeutic efficacy of ARMS-I, mice were challenged with influenza virus as above, and then treated with ARMS-I (10 μL orally) either 4 or 24 hours post infection (hpi), 3 times a day for 5 days. This dosage was chosen to match recommended label usage. Survival and body weight measurements were used to evaluate the effect of ARMS-1, as described above. All experiments were approved by Case Western Reserve University Institutional Animal Care and Use Committee (IACUC) protocol #2011-0200.

**Statistical Analysis:** Pairwise comparisons were performed by a 2-tailed Student's *t* test. The statistical significance between multiple groups was determined by one-way analysis of variance (ANOVA) followed by Tukey-Kramer multiple comparison tests. Kaplan-Meier survival curves were compared using the Mantel-Cox log rank test, followed by pairwise comparisons using the Wilcoxon/Bonferroni's correction for multiple groups. All statistical analyses were performed with Prism, version 6.0 (Graph Pad Software, San Diego, CA). A *P* value < 0.05 was considered statistically significant.

## RESULTS

### 

#### CPC exhibits direct virucidal activity against Influenza A and B virus, including oseltamivir-resistant virus

**Figure 1.**
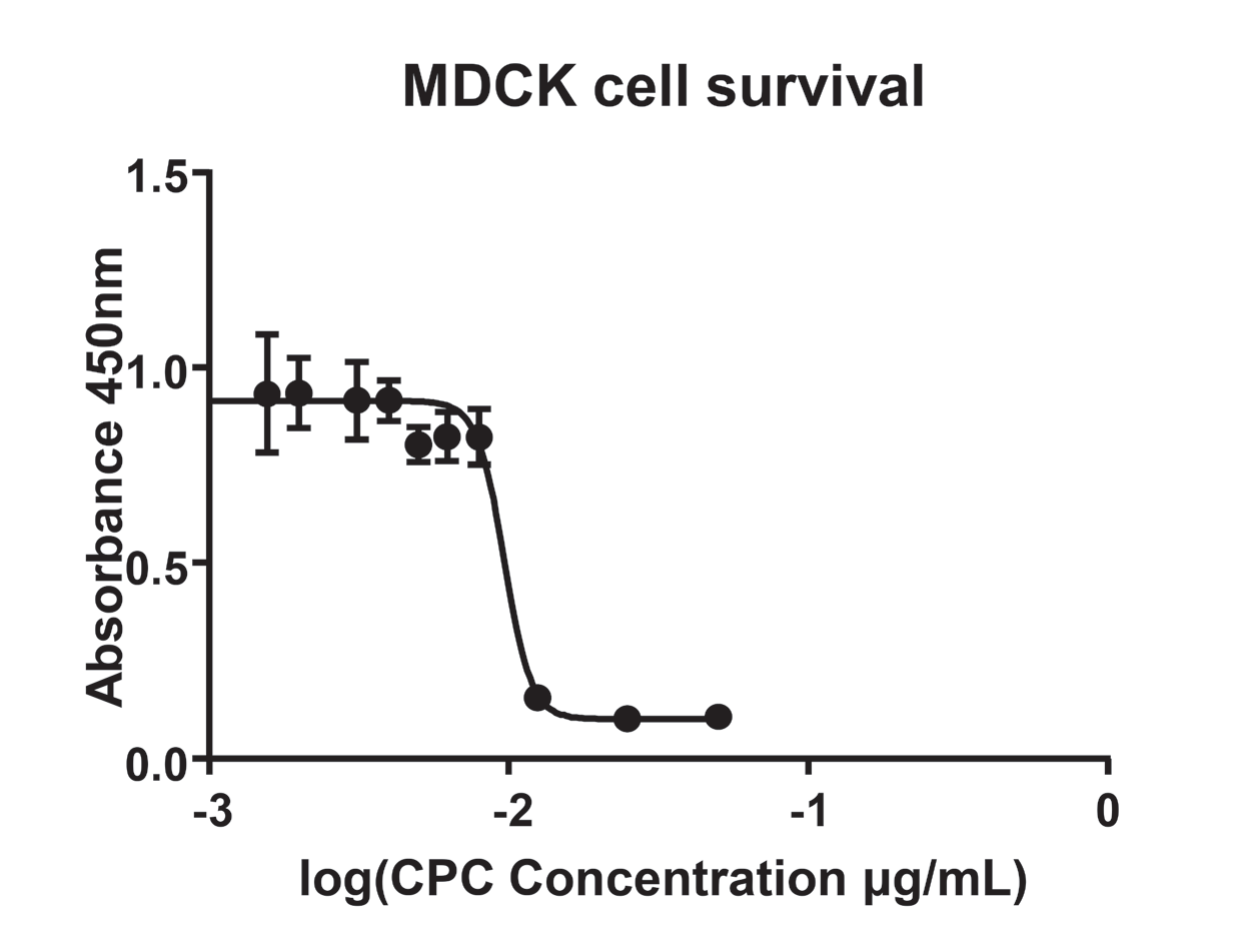
MDCK cell viability following treatment with increasing concentrations of CPC for 10 minutes. The cell viability was determined using the neutral red assay and the absorbance was measured at 450 nm, n = 4 experimental replicates; data represent mean ± SD.

We found that the percentage effective concentration of CPC (EC_50_) against all influenza viruses ranged between 5 μg/mL and 12.5 μg/mL (Table 1) by hemagglutination assay. This was well below the 50% cytotoxic concentration of CPC (CC_50_) of 96 μg/mL (Figure 1) demonstrating protection vs general cell cytotoxicity, confirmed by microscopic visualization. Influenza A required higher concentrations of CPC to reduce the titer by 50% (H1N1:12.5 ± 5.6 μg/mL, H3N2: 10 ± 5.0 μg/mL), whereas influenza B was significantly more susceptible to the disrupting effects of CPC (5 ± 1.9 μg/mL, *P* = 0.001). Based on these data, we determined that the therapeutic index (CC_50_/EC_50_) of CPC ranges between 7.7 and 19.2 (Table 1).

**Table 1.** Effective virucidal concentration (EC_50_) and therapeutic index of CPC against influenza viruses.

Virus (ATCC Strain Number)	EC_50_ (Range)	Therapeutic Index**
Influenza A H3N2	10 **μg/mL (2-17 μg/mL)**	9.6
Influenza A H1N1	12.5 **μg/mL (6-17 μg/mL)**	7.7
Influenza B	5 **μg/mL (2-8 μg/mL)**	19.2
Clinical Isolate 40	6 μg/mL (6 - 6 μg/mL)*	16
Oseltamivir Resistant Influenza A	8 μg/mL (6 - 6 μg/mL)*	12
Oseltamivir Resistant Influenza B	8 μg/mL (6 - 6 μg/mL)*	12

EC_50_ was calculated by hemagglutination assay titer performed with n = 6 experimental replicates. Data represent mean ± SD.

* All experiments were repeated 6 times and resulted in the same value.

** Therapeutic Index was calculated by CC_50_ / EC_50_; CC_50_ was determined to be 96μg/mL by neutral red assay

Hemagglutination assay results were next confirmed with an infectious assay. Specifically, we determined the EC_2Log_ by TCID_50_. Concordant with hemagglutination data, we observed that CPC conferred a 2 log reduction in influenza virus by TCID_50_ (EC_2log_) at ≤20 μg/mL (EC_2log_) for all influenza strains tested. Again, influenza B appeared more susceptible to CPC than influenza A (4 μg/mL vs 20 μg/mL respectively).

To assess if the susceptibility to CPC is different in oseltamivir-resistant influenza strains, we compared the virucidal activity of CPC against oseltamivir-resistant virus to that of oseltamivir-susceptible strains. Our data showed that CPC was effective against both susceptible and resistant influenza strains. Table 1 shows the EC_50_ for resistant influenza A and B viruses were both 8 μg/mL, while the EC_50_ for susceptible isolates ranged between 5 and 12.5 μg/mL. Notably, these EC50 values were all close to one another suggesting that CPC is equally effective against both susceptible and resistant viral strains.

**Figure 2.**
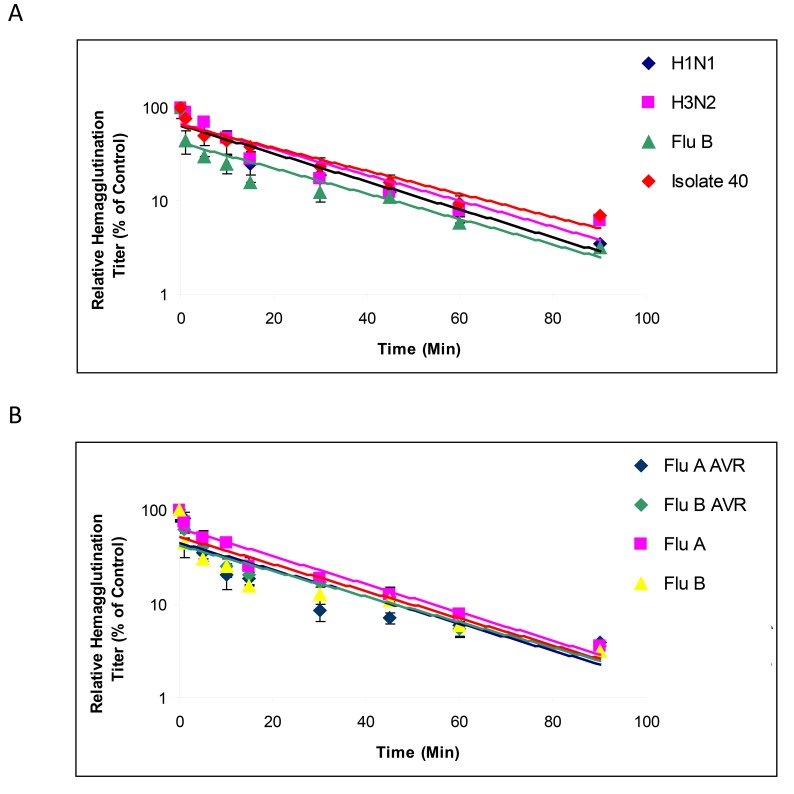
Semi-logarithmic time-kill curves of influenza exposed to EC50 concentrations of CPC at increasing exposure times are shown. The resulting percentage of hemagglutination compared to control is shown over time with mean and standard deviation reported at the indicated time post CPC at EC50. The trend line displays the rate of inactivation for each virus. **A)** Fifty percent of infectious virus was inactivated in the first 5 minutes of exposure for all isolates except for FluB. Isolate 40 is a clinical strain of influenza A (H1N1pdm09) virus propagated from a patient sample. **B)** Time-kill curve between oseltamivir-resistant and susceptible strains. No difference in CPC susceptibility was seen between strains, n = 3 experimental replicates.

Likewise, CPC conferred protection against both susceptible and resistant strains, as measured by time to inactivation (Figure 2B). Virus infectivity was reduced by 50% following 5 minutes of exposure to CPC and 90% reduction at 90 minutes. Taken together, these results indicate that CPC has substantial and rapid (within minutes of exposure) antiviral activity against susceptible and resistant influenza virus.

#### Mechanism of action of CPC against influenza virus involves rapid disruption of the viral envelope

**Figure 3.**
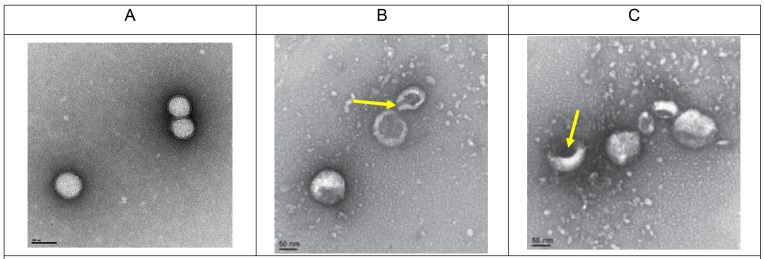
Transmission electron microscopy (TEM) demonstrating CPC disrupts the integrity of the viral envelope and morphology of influenza virus. (A) Untreated influenza virus, (B, C) influenza virus treated with 50 μg/mL CPC for 5 minutes. Viral particles exposed to CPC demonstrate disrupted envelope or cavitation (arrows) of viral units. The presence of negative stain inside the virions indicates membrane permeabilization. The scale bar is in the lower left corner at 100 nm (A) or 50 nm (B, C). We quantified the number of intact and disrupted viruses after treatment, and found that in CPC-treated samples, 86% (172/200) of the viruses were disrupted while in untreated samples only 4.5% (9/200) were disrupted.

The mechanism of action of CPC against influenza virus was evaluated using time-course and TEM analyses. First, influenza virus was exposed to CPC for increasing periods of exposure and the remaining intact virus was titered by HA. Exposure of influenza virus to CPC for more than 5 minutes caused ≥ 50% decrease of viability in all viral strains tested except H3N2 which attained 50% reduction following 10-minute exposure. Moreover, continued exposure to CPC for 90 minutes led to a 90% decrease in influenza titer (Figure 2A). Second, TEM analyses showed that while the viral envelope of untreated influenza viruses was intact, exposure to CPC led to disruption of the envelope and gross distortion of viral ultrastructure (eg cavitation, Figure 3). The presence of negative stain in the interior of virions treated with CPC, but not in untreated virions, suggested permeabilization of the viral membrane. Quantification of the number of intact and disrupted viruses after treatment revealed that 86% (172/200) of the viruses were disrupted or lacked the envelope in CPC-treated samples, while 4.5% (9/200) of untreated virus preparations (in PBS) displayed disrupted or non-enveloped morphologies.

**Figure 4.**
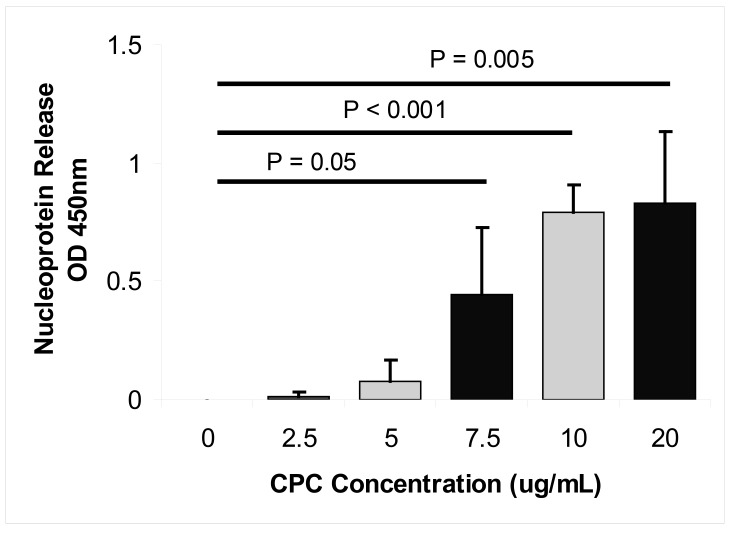
Influenza nucleoprotein release from the virus in response to increasing concentrations of CPC was measured by ELISA. Exposure to CPC concentrations above 7.5 μg/mL produced significant elevations in nucleoprotein release compared to control, n = 8 experimental replicates, shown as mean ± SD. Mean influenza nucleoprotein levels in the presence of CPC were compared to control (0 μg/mL) using independent samples *t* test (IBM SPSS ver 24, IBM Corp).

To further confirm that CPC disrupts the influenza virus envelope, we used ELISA to monitor the release of nucleoprotein from viruses in response to increasing concentrations of CPC. We found that exposure of virus to increasing concentrations of CPC (7.5, 10, and 20 μg/mL) resulted in significantly increased levels of viral nucleoprotein into the media supernatant (*P* ≤ 0.05, Figure 4). The level of released nucleoprotein reached a plateau at CPC concentrations above 10 μg/mL. Taken together, these results demonstrate that CPC acts against influenza virus by rapid disruption of the viral envelope.

#### Exposure to CPC does not induce the development of resistance in influenza virus

**Figure 5.**
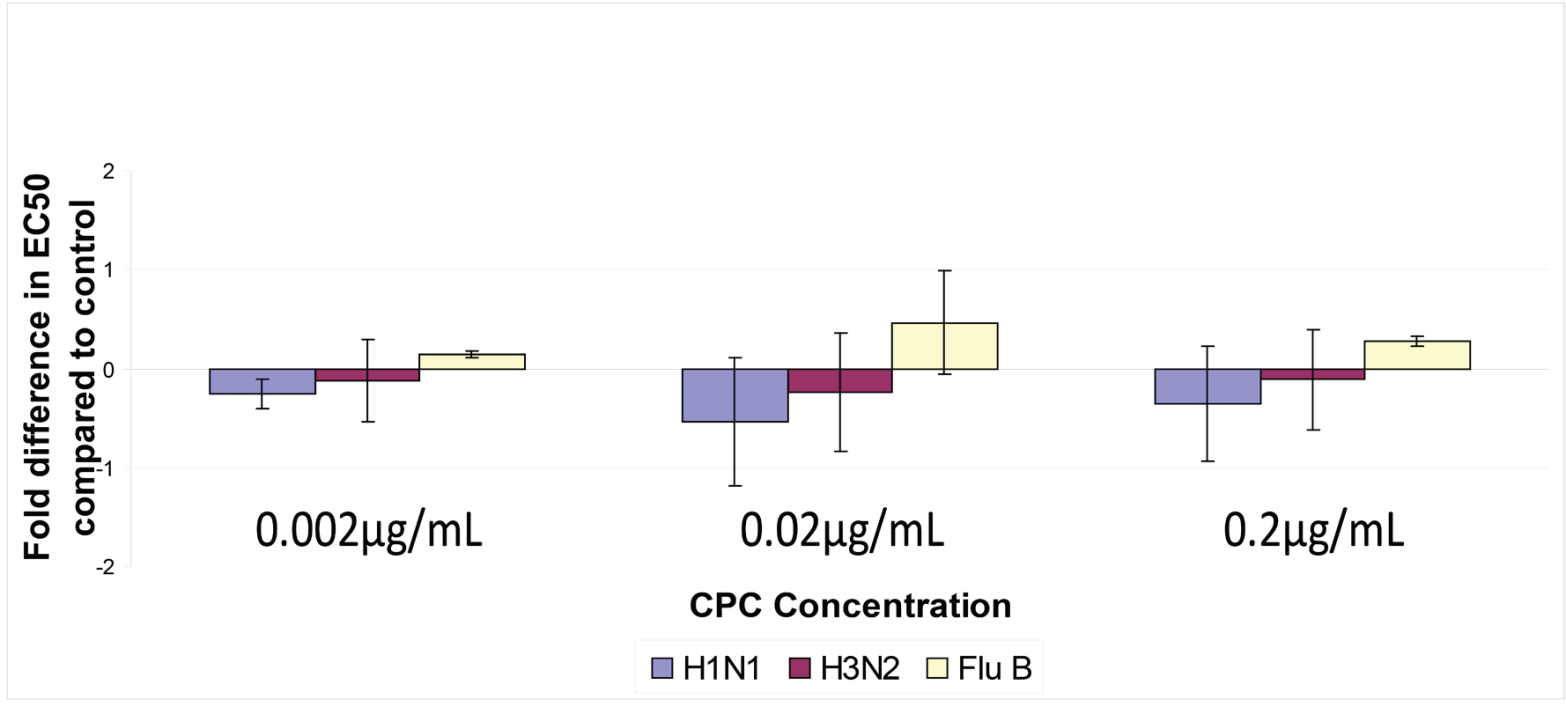
Exposure of influenza to 3 sub-inhibitory concentrations of CPC for 10 passages shows an absence of resistance to CPC. Influenza A (H1N1, H3N2) and Influenza B strains were continuously grown in 3 sub-inhibitory concentrations of CPC: 0.2, 0.02, and 0.002 μg/mL. Viral titer was determined at the end of each passage by hemagglutination assay to ensure an adequate inoculation for each subsequent passage. Ten passages, with 3–4 days exposure per passage, of each influenza strain were performed for all concentrations of CPC. The EC_50_ was then determined for each virus at the 10th passage and compared to the original strain (baseline control). No development of resistance to CPC (defined as over 2-fold change in EC_50_) was seen in any strain of influenza at any tested concentration of CPC, n = 3 experimental replicates, shown as mean ± SD.

To evaluate the potential of CPC to induce drug resistance, influenza virus was exposed to sub-inhibitory concentrations of CPC, and the EC_50_ following 10 passages of drug exposure was determined (Figure 5).

We defined CPC resistance as EC_50_ values > 2-fold the mean EC_50_ between treatment and control. This definition is more stringent than that used for other influenza medications [35].

No increase in EC_50_ was noted at any concentration for either H1N1 or H3N2. Influenza B demonstrated a slight increase in EC_50_ when exposed to CPC for 10 passages that was not significant. Of note, influenza B susceptibility to CPC continued to be comparable to that of influenza A. These results suggested that CPC has a low potential to select for influenza virus resistance consistent with its mechanism of action.

#### Orally applied CPC formulation exhibits prophylactic and therapeutic efficacy against influenza infection in a murine model

The clinical formulation of CPC named “ARMS-1” is used prophylactically for upper respiratory infections. In a randomized, double-blind clinical trial, we demonstrated that this topical oral CPC formulation was well-tolerated and reduced the severity and duration of cough and sore throat in the enrolled study participants vs the control group [34]. Although clinical efficacy met statistical significance, this study was not powered to address efficacy against specific pathogens (ie, influenza). To determine the physiological relevance of CPC against influenza, we evaluated the efficacy of the CPC-based clinical formulation ARMS-1 containing 0.1% CPC w/v as the active ingredient [34]. We tested whether ARMS-1 conferred protection from the widely used mouse-adapted influenza H1N1 strain PR8 [36] in both prophylactic and therapeutic models of influenza infection.

**Figure 6.**
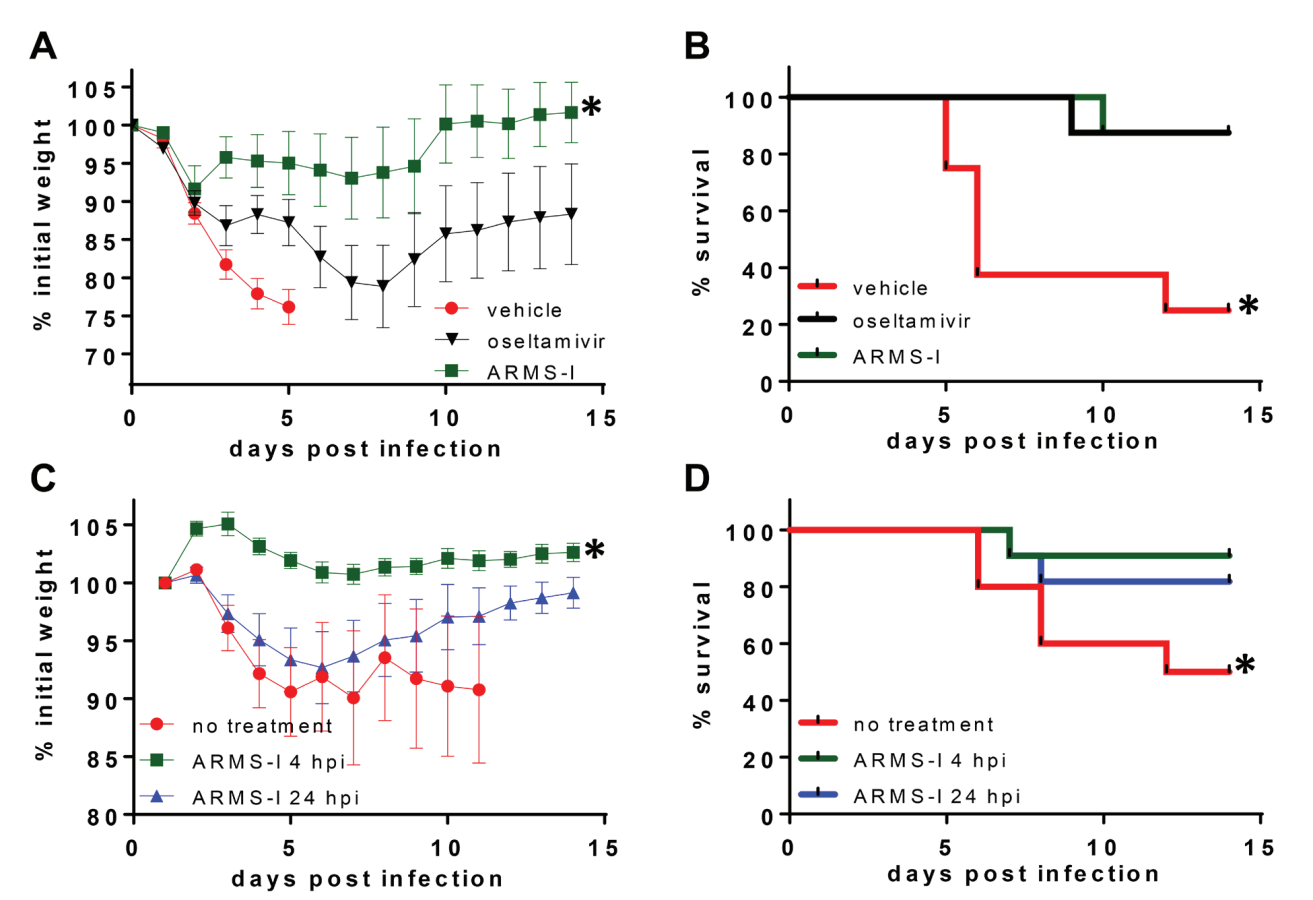
CPC formulation (ARMS-I, 0.1% CPC w/v) reduced influenza-associated pathogenicity *in vivo*. ARMS-I prophylactic protection from morbidity and mortality was demonstrated by (A) body weight (percentage of initial weight on Day 0) and (B) Kaplan-Meier survival curve, respectively. The ARMS-I and oseltamivir groups were treated 15 minutes before the challenge and then twice a day for 5 consecutive days.

Evaluation of the prophylactic efficacy of ARMS-I showed that body weights of PBS-treated mice were significantly reduced at 3, 4, 5, and 6 days post infection (dpi), compared to ARMS-I treated mice (*P* ≤ 0.046, Figure 6A). The increase in relative body weight between ARMS-I-treated and untreated mice ranged between 14% on day 3 and 24.8% on day 6 post-infection. Of note, ARMS-I-treated animals tended to exhibit higher body weights than oseltamivir-treated mice, with a significant difference noted on day 3 (95.7% vs 86.8%, respectively; *P* = 0.033). Increased weight in ARMS-I-treated animals was consistent with increased activity (decreased morbidity) noted during daily observation. Survival analysis demonstrated that ARMS-I-treated mice exhibited significantly increased survival compared to untreated mice *(P* = 0.0102, Figure 6B). There was no significant difference in survival between the ARMS-I-treated vs oseltamivir-treated groups.

Given the promising protection observed prophylactically, we next asked whether ARMS-I might be protective *after* infectious challenge. We found that animals treated 4 hpi with ARMS-I did not have significant weight loss in contrast to animals which were untreated or treated 24 hpi and exhibited up to 10% reduction in body weight, during the first 7 days (Figure 6C). Survival analysis demonstrated that ARMS-I treatment 24 hpi significantly increased survival compared to untreated mice *(P* = 0.047, Figure 6D). Mice treated 24 hpi with ARMS-I also had increased survival at the termination of the experiment (14 dpi). However, this difference was not statistically signifi-cant *(P* = 0.08, Figure 6D).

These *in vivo* studies provide physiological evidence that CPC-based treatments may be efficacious in the prevention and treatment of influenza-associated morbidity and mortality.

Similarly, ARMS-I therapeutic protection was demonstrated when given 4 or 24 hours *post* infection by (C) body weight (percentage of initial weight on Day 0) and (D) Kaplan-Meier survival curve. Weights were not reported after > = 50% of mice died. Asterisk (*) indicates *P* < 0.05 between indicated ARMS-1 and either vehicle or no treatment group in prophylactic (A, B) and therapeutic (C, D) studies, respectively, n = 8 to 10 mice/group. One of 2 similar experiments is shown

## DISCUSSION:

We found that CPC is active against susceptible and oseltamivir-resistant influenza strains. CPC disrupted influenza particles rapidly, within minutes of exposure, analogous to CPC's effects on other pathogens [29]. Viral resistance was not observed after prolonged exposure over multiple passages *in vitro*, consistent with the physicochemical mechanism of action of CPC. This study demonstrates that CPC can directly disrupt the membranes and subsequently inhibit infection of pathogenic influenza viruses. Lastly, we found that the clinical formulation of CPC reduced influenza-associated morbidity and mortality *in vivo.* These findings have significant implications for the prevention of influenza.

Cetylpyridinium chloride is one of the more active quaternary ammonium compounds (QACs) but, despite its use in the food, pharmaceutical, and medical industries for over fifty years, there is little data specifically detailing its mechanism of action against viruses. It is assumed that CPC has direct action against lipid bilayer membranes, particularly the cytoplasmic membrane, leading to leakage of cytoplasmic contents and lysis of the microbial cell [29, 37]. This mechanism allows for a broad but varied spectrum of activity among different types of microorganisms with bacteria, fungi, and enveloped viruses such as HIV described as highly susceptible [27, 38]. There have been no studies published to date on the effectiveness of CPC against respiratory viral pathogens such as influenza. Here we demonstrate that the EC_50_ of CPC against influenza viruses is within a clinically meaningful range.

All influenza strains had CPC-associated EC_50_ and EC_2log_ well below the CC_50_ of MDCK cells consistent with a clinically viable therapeutic index. Interestingly, influenza B had lower EC_50_ and EC_2log_ compared to influenza A suggesting that this strain is more susceptible to the effects of CPC. The reasons for this remain unclear as both influenza A and B derive their envelope from the host cell. However, reports have shown that the phospholipid composition of purified virions differs from that of the host cells. Whereas phosphatidylcholine is the major component in most mammalian cell membranes, in purified virions phosphatidylethanolamine dominates. In addition, analysis of lipid species of the viral envelope revealed subtle differences between influenza A and B strains [39]. As these bilayers are the target for QACs, such differences may lead to an alteration of CPC affinity resulting in changes of susceptibility to CPC.

The ability of CPC to perturb viral envelopes allows for a broad spectrum of activity. However, historical concerns over the toxicity of CPC may have resulted in it being underused clinically. In our study, the therapeutic index (EC_50_/CC_50_) of CPC was between 7.7 and 19.2, within the range for consideration of drug development. However, it is important to note that MDCK cells are quite robust; further cytotoxicity testing of CPC in human-derived cells, such as A549 cells, may add to our understanding of potential toxicity. We expect that longer exposure may also portend increased toxicity. Importantly, CPC has been deemed safe for human use when applied at concentrations up to 1000 μg/mL [25, 27, 28]. Additionally, extensive safety studies have already been conducted with CPC, including animal pharmacokinetic absorption, distribution, metabolism and excretion (ADME), carcinogenicity, developmental toxicity, and reproductive toxicity studies [25, 26, 28]. These studies were conducted in a wide array of animal models (mice, rats, hamsters, rabbits, cats, and dogs) and humans demonstrating that CPC is safe for human use at the doses used in this study, including the 0.1% w/v clinical formulation. Lastly, a randomized double-blind clinical trial demonstrated that the CPC formulation (ARMS-I) was safe, well tolerated, and had a high acceptability with a protective signal [34].

The virucidal kinetics of CPC against influenza viruses sensitive or resistant to oseltamivir were similar to the rapid antimicrobial activity described for bacteria and fungi [29]. Similar findings were reported by Maillard *et al* who found that disruption of bacteriophage F116 structure occurred within minutes following CPC exposure [40]. For prevention of viral invasion and development of disease, CPC with its broad spectrum and rapid virucidal activity may be preferred.

Our TEM and nucleoprotein release studies showed that CPC disrupts the viral envelope. Although these experiments were performed using influenza B as a prototype virus, we expect the results to be similar with influenza A. This observation is in agreement with the known mechanism of action of CPC. Importantly, it is likely that CPC would also have substantial activity against other enveloped viruses such as RSV, parainfluenza, and coronavirus. The common annual epidemic of these viral pathogens in addition to influenza highlight the unmet need for broad anti-virals. The potential activity of CPC against non-enveloped pathogens including rhinovirus, bocavirus, or adenovirus remains unclear. However, since CPC has been shown to cause structural damage in non-enveloped bacteriophage [40], this agent may have activity against non-enveloped human viral pathogens as well. Thus, further evaluation of CPC effectiveness against viral pathogens other than influenza, both enveloped and non-enveloped, has potential clinical value.

Overuse of NAIs throughout the world has quickly led to the spread of neuraminidase resistance [41]. In contrast, QACs, including CPC, have been actively deployed since the 1930s with no apparent reduction in their effectiveness against bacteria and fungi [29]. The reason that resistance to CPC has not been observed is likely attributable to its mechanism of action, which involves binding to the microbial lipid bilayer, and CPC acts against influenza by disrupting the lipid envelope, which is analogous to the activity against bacteria and fungi. Since the viral envelope is host derived, the physicochemical mechanism of CPC is independent of intrinsic viral proteins and unlikely to be influenced by mutation. We have shown that influenza strains had no significant increase in EC_50_ after 10 passages in the presence of sub-inhibitory CPC concentrations. This lack of resistance potentiation by CPC contrasts with the dramatic development of oseltamivir resistance after as little as 4 to 6 passages under similar conditions [42, 43]. In this regard subtherapeutic oseltamivir exposure can result in > 1000-fold increase in IC_50_ [43, 44]. However, continued exposure of influenza to CPC beyond 10 passages should be performed to ensure that there is no late development of resistance.

Lastly, we demonstrated protection conferred by the CPC-based ARMS-1 clinical formulation in a mouse model of influenza. Often, drug candidates possess potent *in vitro* inhibitory activity but fail when tested *in vivo*. We found that mice treated with ARMS-1 either before or following infectious challenge exhibited significantly increased survival compared to vehicle control mice and comparable to oseltamivir-treated mice. In addition, no toxicity was detected in the mice treated with ARMS-1 in our experimental mouse model. In fact, the ARMS-1 treatment group had higher weights and normal activity when compared with untreated, PBS-treated and oseltamivir-treated groups. This protection was most prominent when ARMS-1 was given either 15 minutes before or 4 hours after infection. Both of these time points are prior to clinical symptoms. These findings support prophylactic use and indicate that a CPC-based treatment would probably not work after infection is established. Future studies are needed, including similar *in vivo* trials in ferrets, another important animal model for influenza infection, to assess the potential of CPC-based therapies to protect against influenza disease.

In conclusion, we demonstrated that CPC shows significant activity against influenza via direct virucidal activity by disruption of the viral envelope and no indication of resistance selection. Increased survival was observed in mice treated with the CPC formulation, compared to untreated mice, a finding similar to the anti-influenza agent oseltamivir. We submit that CPC has the ability to protect against influenza respiratory tract infections and should be considered for further clinical development.
